# Application of high-dimensional feature selection: evaluation for genomic prediction in man

**DOI:** 10.1038/srep10312

**Published:** 2015-05-19

**Authors:** M. L. Bermingham, R. Pong-Wong, A. Spiliopoulou, C. Hayward, I. Rudan, H. Campbell, A. F. Wright, J. F. Wilson, F. Agakov, P. Navarro, C. S. Haley

**Affiliations:** 1 MRC Human Genetics Unit, MRC Institute of Genetics and Molecular Medicine, University of Edinburgh; 2 The Roslin Institute and Royal (Dick) School of Veterinary Studies, University of Edinburgh; 3 Centre for Population Health Sciences, University of Edinburgh; 4 Pharmatics Limited, UK

## Abstract

In this study, we investigated the effect of five feature selection approaches on the performance of a mixed model (G-BLUP) and a Bayesian (Bayes C) prediction method. We predicted height, high density lipoprotein cholesterol (HDL) and body mass index (BMI) within 2,186 Croatian and into 810 UK individuals using genome-wide SNP data. Using all SNP information Bayes C and G-BLUP had similar predictive performance across all traits within the Croatian data, and for the highly polygenic traits height and BMI when predicting into the UK data. Bayes C outperformed G-BLUP in the prediction of HDL, which is influenced by loci of moderate size, in the UK data. Supervised feature selection of a SNP subset in the G-BLUP framework provided a flexible, generalisable and computationally efficient alternative to Bayes C; but careful evaluation of predictive performance is required when supervised feature selection has been used.

Genome-wide association studies (GWAS) have successfully identified a large number of single nucleotide polymorphisms (SNPs) associated with complex traits^1^. These GWAS hits have not only increased the understanding of the underlying physiology, but also information from these SNPs hold the promise of more accurately predicting the phenotypes of complex traits. These predictions could underpin health management in humans and genomic selection in animal and plant populations in the near future^2^. Prediction using GWAS hits has not, however, been very successful^3^. This is because the variants identified from GWAS explain only a fraction of the total genetic variation^4^. The analysis of effects of many SNPs jointly is gaining attention, as each SNP may account for a low portion of phenotypic variance^5^, but jointly large numbers of SNPs may capture a larger proportion. The risk of overfitting is high when using high dimensional genomic data. Feature selection techniques are designed to identify SNPs associated with complex traits^6^. By selecting a reduced number of SNPs with significantly larger effects compared to other SNPs, researchers can focus on the most promising SNPs for use in genomic prediction. The reduced dimensionality provides for better generalisation due to a lower number of model parameters to be estimated from the data. Despite the importance of reducing dimensionality, only a few studies have used feature selection methods on GWAS data^7^,^8^,^9^. Furthermore, when analysing genome-wide genetic variants, one also has to consider that some regions of the genome may be overrepresented due to elevated levels of linkage disequilibrium (LD), diluting the genome-wide patterns that reflect ancestry differences^10^, which has not been considered in feature selection algorithms for genomic prediction to date. In the current study, we will develop an unsupervised and four supervised features selection approaches to accommodate the inherent structure of GWAS data by incorporating information from established genetic epidemiologic methods^11^,^12^; to generate the best reconstruction of the data to provide more accurate predictions.

Several whole-genome regression methods have been proposed and evaluated that regress phenotypes on whole-genome markers simultaneously^13^, following the ground-breaking contribution of Meuwissen *et al.* (2001). The list of available methods that allow implementation of whole-genome regression is long; including Bayesian regression^2^ and genomic best linear unbiased prediction (G-BLUP) from animal breeding and ridge regression, least absolute shrinkage and selection (LASSO), and the elastic net, support vector regression (SVR)^13^, graphical Gaussian models (GGMs)^14^ and sure independence screening (SIS)^15^ from machine learning. In this study we will focus on two of the most commonly used methods from animal breeding, including Bayesian regression and G-BLUP^13^. G-BLUP is attractive because its implementation is straightforward using existing BLUP software. This approach yields estimates equivalent to those from ridge regression^16^. The Bayesian methods have been discussed and described widely, and are generally chosen because many of them allow departures from the infinitesimal model in which very many quantitative trait loci (QTL) each have a very small effect^16^. Bayes C, is a modification of the original Bayes B approach described by Meuwissen *et al.*^2^ in which the proportion of SNP with zero effects is estimated from the data^17^. In G-BLUP all marker are assumed to explain equal variance, while the Bayes C approach allows markers to explain different amounts of variation, and in the case of oligogenic traits, assign a small number of markers to have an effect and many markers to have no effect^13^,^18^. In this study we will compare the predictive performance of G-BLUP and Bayes C with and without feature selection.

Feature selection is a process in which subsets of available features are selected for application in prediction models. The best subset of features contains the least number of dimensions that most contribute to prediction accuracy^19^. Feature selection is separate and different from model evaluation. It is therefore important to ensure that predictive models are evaluated on data that has not been used for estimating model parameters (training). This is commonly achieved by withholding a subset of data for testing once or repeatedly (e.g. in cross-validation). Unfortunately, in some prediction studies there has been a tendency to select markers associated with an outcome using the complete dataset^20^,^21^,^22^. As we will demonstrate later in this paper, this may lead to a significant bias in the estimates of the predictive accuracy.

Trait heritability and genetic architecture have been shown to influence prediction accuracy^13^. Three traits were therefore selected in this study based on their different genetic architectures. Height is highly heritable, has been thoroughly studied in humans and is often used as an example of highly polygenic trait^23^. Typically, the heritability of height is estimated as 80%^24^. A genome wide meta-analysis (GWMA) identified 180 loci^25^ harboring genetic variants associated with height. Epidemiologic studies have revealed that a low plasma concentration of high density lipoprotein (HDL) cholesterol is a potential risk factor for coronary heart disease. HDL levels are also strongly influenced by genetic factors. The heritability from several family and twin studies has been estimated to range between 40 and 60%^26^. HDL is influenced by few loci of moderate effect as well as a large number of loci with small effects, and a recent GWMA that identified 38 genome-wide significant loci^27^ that influence plasma HDL concentration. Obesity is a major public health issue, resulting in increased morbidity and mortality and huge healthcare and economic burden^28^. Body mass index (BMI) is the most commonly used metric for adiposity. Heritability studies have shown that genetic factors contribute 40–70% of the population variation in BMI^29^. A large scale GWMA has identified 18 loci associated with BMI^30^. The few identified loci have small effects and the trait seems influence also by a large number of loci with even smaller effects that cannot be detected in current GWMA^31^. The three traits provide an opportunity to investigate how different trait architectures influence the accuracy of prediction under various feature selection scenarios and prediction methods.

## Results

### Population stratification

Correction for population stratification by fitting the top ancestry principal components (PCs) in G-BLUP had only a marginal effect on accuracy of prediction within the Croatian, while improving predictive performance in the ORCADES (UK) replication data (see Supplementary Results 1). The top 24 PCs were therefore used to correct for stratification in G-BLUP, and while computing GWAS or conditional P-values for supervised feature selection in this study.

### Genomic heritability

The genomic heritability estimates were very close to 0.8, 0.3, and 0.5 for height, BMI and HDL, respectively in Croatian and Croatian-ORCADES combined data sets (Supplementary Table 1). These heritability estimates were consistent with posterior distribution of these parameters in this data within the Bayes C framework (Table 1). The heritability estimates across the ten training data sets were comparable to the estimates from whole Croatian data set (Supplementary Table 1).

### Prediction accuracy

Bayes C achieved the highest accuracy for the prediction of height and HDL within the Croatian dataset (Table 2). The accuracies obtained from G-BLUP and Bayes C for the prediction of BMI within the Croatian data were similar. However, when predicting height and BMI into the ORCADES data set, G-BLUP achieved the highest accuracy. The Bayes C and the meta-analysis hits QTL linear regression analysis model attained similar accuracies for the prediction of HDL into the ORCADES data.

In this study, the theoretical expectation of accuracy based on the heritability estimates (95% confidence intervals) from the entire Croatian dataset in this study (Supplementary Table 1) are 0.44(0.42-0.46), 0.33(0.29-0.37), 0.29(0.25-0.33) for height, HDL and BMI respectively in the Croatian data. In the ORCADES replication dataset the theoretical expectation for accuracy of prediction for height, HDL and BMI are 0.13(0.12-0.13), 0.10(0.09-0.11) and 0.09(0.07-0.10) respectively^13^. The estimates of prediction accuracy obtained with G-BLUP and Bayes C within the four Croatian datasets were less than the theoretical expectation and in the order of 0.25, 0.19 and 0.12 for height, HDL and BMI respectively (Table 2). The estimates of prediction accuracy into the ORCADES data were much lower than theoretical expectation, irrespective of the model used. However, when individuals from the ORCADES data were used to train the G-BLUP model, there was a substantial improvement in prediction accuracy for all three traits within the ORCADES data and the estimated accuracies for height, HDL and BMI within the ORCADES data were close to theoretical expectation (Supplementary Table 2).

### Feature selection

**Figure 1:**
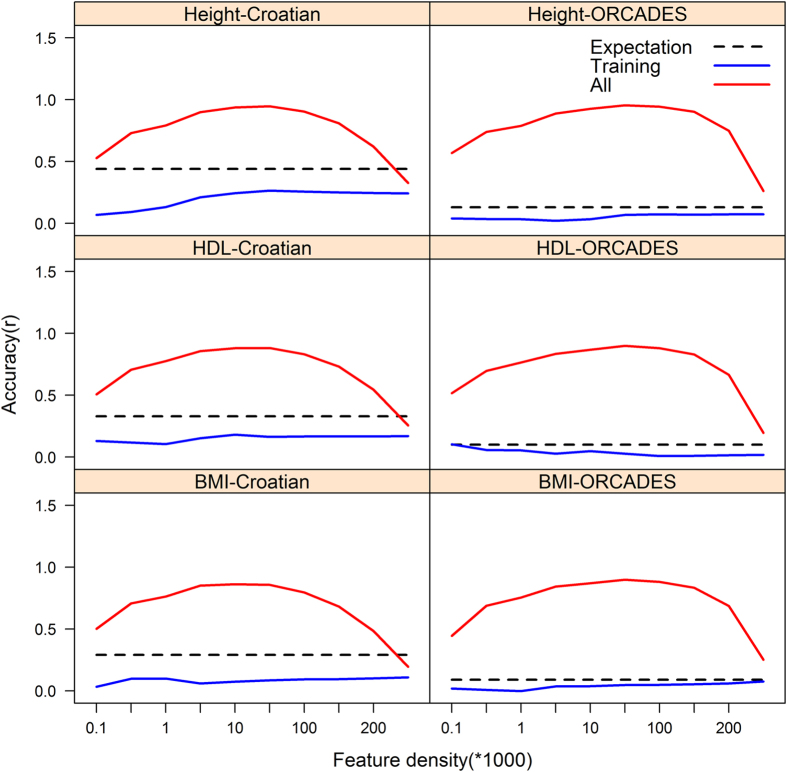
The mean prediction accuracy (correlation between predicted and observed phenotype) across the test data sets, following tenfold cross validation the Croatian data and into ORCADES replication data; when feature subsets were ranked, and subsequently selected based on GWAS p-values estimated in each of training folds (“Training”), and when feature subsets were ranked, and subsequently selected based on GWAS p-values estimated from the whole Croatian data set (“All”). The broken black lines depict the theoretical expectation (Expectation) in related and unrelated individuals^13^. The sold blue lines depicts the mean accuracy results across the folds when ranking and selection of feature subsets was based on GWAS P-values estimated from the training data only. The sold red lines depicts the mean accuracy results across the folds when ranking and selection of feature subsets was based on GWAS P-values estimated from all the Croatian data. There was substantial inflation of the prediction accuracy for all three traits in this study, when training data was used in feature selection i.e. when subsets were ranked and subsequently selected based on GWAS p-values estimated from the whole Croatian data set.

There was substantial inflation of the prediction accuracy for all three traits in this study, when validation sets were previously used in feature selection i.e. when subsets were ranked, and subsequently selected based on GWAS p-values estimated from the whole Croatian data set. The prediction accuracy exceeded theoretical expectation by up to 2 fold for all three traits (Supplementary Table 3). Failure to cross-validate the selected features resulted in even greater upward bias when predicting into the ORCADES replication data. Prediction accuracy exceeded theoretical expectation by up to 7, 8, and 9 fold for height, HDL and BMI respectively (Fig. 1; Supplementary Table 3). The proportion of individuals in the test data had no substantial impact on the upward bias in prediction accuracy when the features were not cross-validated (Supplementary Fig. 1). In all subsequent supervised feature selection scenarios, feature subsets were selected solely on the basis of the training data without using data from the test sets.

**Figure 2:**
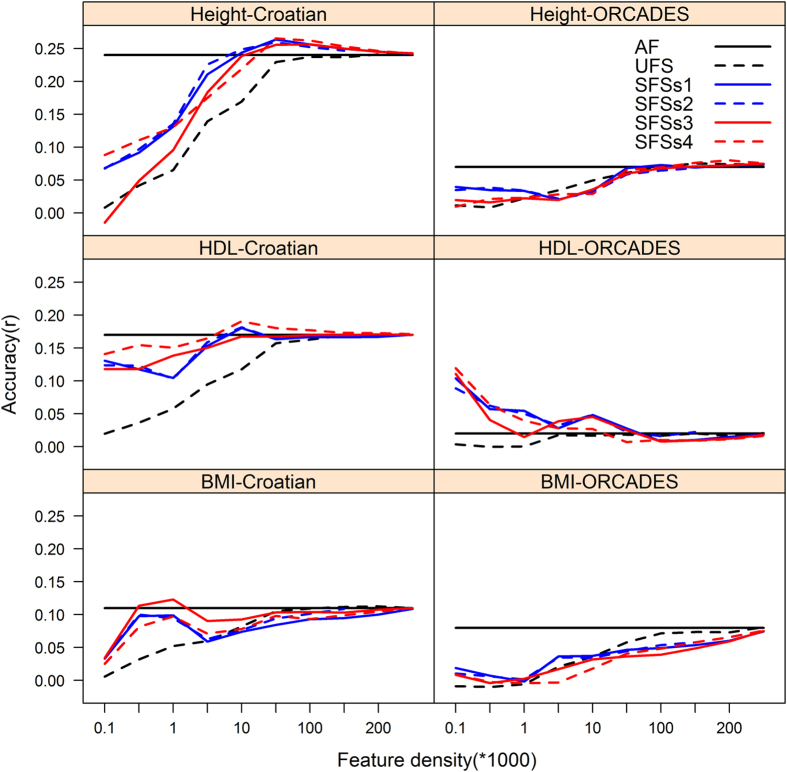
Average prediction accuracy (correlation between predicted and observed phenotype) across the test data sets, following tenfold cross validation the Croatian data and into the ORCADES replication data using the different marker densities selected using unsupervised, and four supervised methods of feature selection in the GBLUP frame work. The solid black line depicts the accuracy results from the full feature set of 263,357 markers. The broken black depicts the accuracy results across the different feature subset densities following unsupervised feature selection (UFS). The solid blue and broken blue and solid red, broken red lines depicts the accuracy results across the different feature subset densities following supervised feature selection scenarios (SFSs) 1-4: 1) feature selection based on ranking of trait specific genome wide association (GWAS) P-values; 2) feature selection based on ranking of trait specific GWAS P-values, and pruning based on median SNP distance (MSD) in the data this study ; 3) feature selection based on ranking of trait specific GWAS P-values, and re-ranking based on MDS conditional P-values, and 4) feature selection based on ranking of trait specific GWAS P-values, and re-ranking based on haplotype-block specific conditional P-values, respectively. The four supervised feature selection methods performed similarly. The best performance was obtained by using a small, intermediate, or large number of SNPs in the predictive models; depending on the trait architecture and/or whether the feature selection approach was supervised or not.

**Figure 3:**
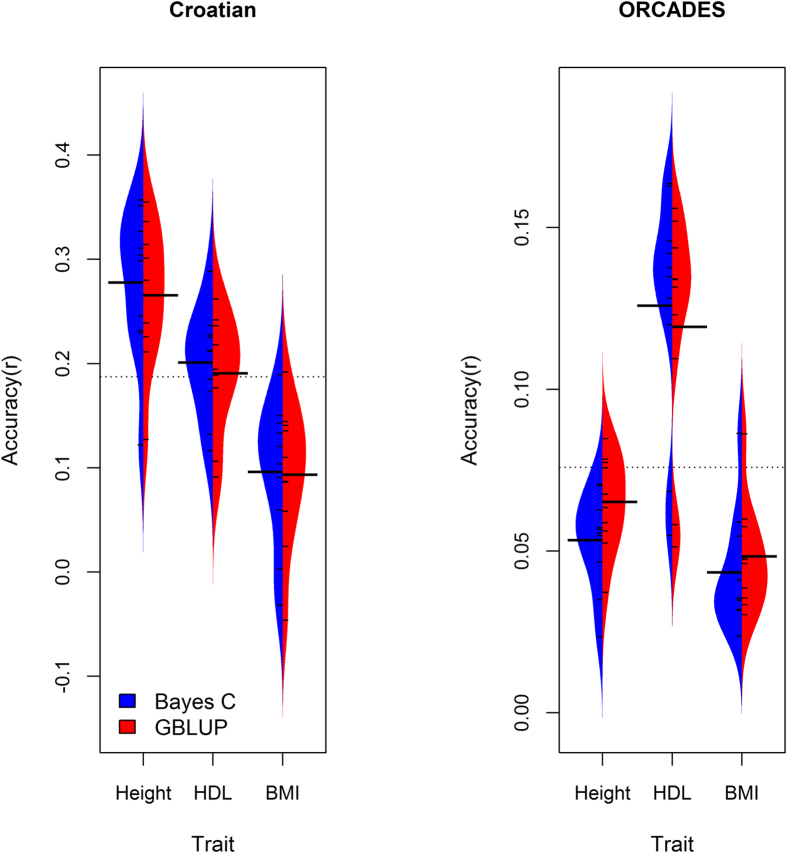
Distribution of prediction accuracy (correlation between predicted and observed phenotype) across the test data sets, following tenfold cross validation the Croatian data and into ORCADES replication data from the non*-*redundant subsets for height, high density lipoproteins (HDL) and BMI selected using supervised feature selection methods based on ranking based on haplotype-block specific conditional P-values in Bayes C and G-BLUP frameworks. The non*-*redundant feature subsets densities were 50,000 for height and 100,000 for BMI in both datasets, and 10,000 and 100 for high density lipoproteins (HDL) in the Croatian and ORCADES replication data respectively. The Bayes C results are plotted to the left of the center of each plot with a blue distribution and black whiskers; G-BLUP results data are plotted to the right of the center in each plot and shown with a red distribution and black whiskers. The mean of each distribution is given as a long black line. Prediction accuracy results are given relative to mean accuracies across the three traits (broken grey line). Supervised feature selection allowed G-BLUP to achieve equivalent prediction accuracy to Bayes C irrespective of the genetic architecture of the three traits under study.

In this study we compared five different feature selection algorithms with respect to prediction accuracy in 10 fold cross-validation. Furthermore we compared the derived models in terms of the numbers of features required to maximize prediction accuracy. The unsupervised method based on subsets of regressors showed the poorest performance in terms of the number of features required to achieve accuracy analogous to that acquired from the full set of features (Fig. 2). However, the unsupervised method achieved accuracy close to that acquired from the full set of features at lower SNP densities than the supervised methods in the prediction of BMI into the ORCADES data. This method did not improve prediction accuracy above that achieved from the full set of features (Fig. 2). In general, the supervised feature selection scenarios performed similarly. The supervised feature selection method in scenario 4, which ranked SNPs based on haplotype-block-based conditional P-values generally performed as well or better than other methods (Fig. 2) and we therefore evaluated this method in the Bayes C framework. Following supervised feature selection, the Bayes C framework did not provide substantial improvement in prediction accuracy over that observed in the G-BLUP framework (Supplementary Fig. 2; Supplementary Table 4). The distribution of the prediction accuracies across the ten folds estimated using the selected non-redundant feature subsets in the Bayes C and G-BLUP frameworks were broadly overlapping across the three traits in the Croatian and ORCADES replication data (Fig. 3).

## Discussion

The major contributions of this paper are threefold. Firstly, we have demonstrated that feature selection in the G-BLUP frame work provides a more efficient alternative to the computationally time-consuming Bayes C, for traits with few loci of moderate effect size like HDL. Secondly, that supervised outperformed unsupervised feature selection in the prediction of all three traits under study. Finally, failure to cross-validate features, in supervised feature selection produced overoptimistic estimates of prediction accuracy.

The characteristics of the two methods used to fit the genomic prediction models in this study differed. The main advantage of G-BLUP over Bayes C is a significantly lower computational cost (see Supplementary Results 2). The accuracy of prediction is dependent on the proportion of phenotypic variation due to genetic effects^32^. For example, prediction performance for height was the best among the three phenotypes. This is consistent with the magnitude of heritability of height. The estimated heritabilities of 0.8, 0.5, and 0.3 for height, HDL and BMI respectively, are of the same order of magnitude as those reported in the literature^24^,^33^. The G-BLUP and Bayes C models capture three sources of genetic signal: population stratification, family structure and LD between markers and QTL in the training data. Prediction accuracy arises from two of these three sources (i) SNPs in useful LD with causal loci; and (ii) SNPs reflecting the relationship structure between the set of individuals to be predicted^34^,^35^,^36^. The sources of prediction accuracy have bearings on predictive performance across different trait architectures and on how genetic signal is transferred into populations of different ancestry^34^,^35^,^36^,^37^. The sharp decline accuracy observed in this study when predicting height and BMI into the ORCADES data means that the primary source of prediction accuracy within the Croatian data was due to genetic relationship information^36^. This is because traits with genetic architecture approximating an infinitesimal model generate high resemblance between relatives, but weak LD signal between QTL and markers in limited sized data sets^31^. G-BLUP is more effective at capturing genetic relationships; because it fits more markers into the prediction model than Bayes C. In accord, G-BLUP was more effective than Bayes C^13^,^16^,^36^ in the prediction of height and BMI traits into the ORCADES data. On the other hand, when predicting into populations; capturing LD between marker and QTL will be useful because the individuals are of different ancestry. The accuracy observed when predicting into the ORCADES best approximates the accuracy due to LD^34^. In this instance, Bayes C is more effective at capturing LD between markers and QTL than G-BLUP^13^,^16^,^36^,^37^,^38^, as seen for the prediction of HDL with is influence by loci of moderate effect size in this study. The results from this study support the results from theoretical, simulation and empirical genomic prediction studies in man and animals^2^,^9^,^13^,^16^,^34^,^36^,^37^,^38^.

It has been argued that differences between G-BLUP nonlinear Bayesian methods^39^, such as Bayes C will become more pronounced with the availability of full genome sequence data^39^. This is because of the severe shrinkage imposed on individual SNP effects in G-BLUP^40^. In accord, the additional gain from simply using dense, as opposed to full sequence information in G-BLUP is often small (two or three percent)^41^. Nevertheless, in this study we have demonstrated that supervised feature selection allowed G-BLUP to concentrate on the biologically relevant genomic regions; instead of spreading the effects over all SNP across the genome, allowing G-BLUP to achieve an equivalent predictive to Bayes C irrespective of the trait architecture. Thus the application of G-BLUP on sequence data should not be ruled-out.

As is the case in this study, a number of recent genomic prediction studies from animal breeding have also attempted to increase predictive power by combining data from multiple-related populations^42^. While accounting for population substructure and cryptic relatedness is standard in GWAS^10^, genomic prediction studies in admixed populations have so far ignored population-specific effects^9^,^43^,^44^. In this study, accuracy for all traits was marginally lower when predicting within the Croatian data following PC adjustment. This is because there was little genetic differentiation observed among the four Croatian populations (Fst 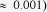
in this study, and standard G-BLUP is known to gain power over PC adjustment, when analysing population which are homogenous for environmental exposures^45^. A greater degree of differentiation was observed between the Croatian and ORCADES populations (Fst 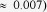
16. Accordingly, the accuracy of prediction in the ORCADES was notably better following PC adjustment. This is because fitting the first few PC from the IBS can reduce the statistical noise^45^ arising from heterogeneity of causality and population stratification^46^. On the basis of these results we recommend that one should account for account for population substructure in G-BLUP.

In this study we have demonstrated that data used for testing should not be used for feature selection and parameter estimation. A number of prediction studies have previously discussed this issue^19^,^20^,^21^. However, here we have demonstrated the importance of setting aside an independent test dataset, and using the remaining data for performing feature selection and model training in genomic prediction in human populations. Otherwise, as observed in this study, one runs the risk of reporting a grossly over-optimistic estimate of the prediction accuracy^19^. We have also demonstrated that the proportion of individuals in the test data that were used for performing feature selection has no impact on the upward bias in prediction accuracy^47^. It is the proportion of the training samples that are also in the Croatian data set used to inform feature selection that drives inflation in the accuracy of prediction; implying the participants that constituted 4% of the meta-analysis population^25^ analysed in the GWAS used to inform feature selection^9^ could have potentially resulted in upward bias in accuracy reported in this current study.

The results of this study indicate that, even with a modest number of cross-validated molecular markers, G-BLUP and Bayes C prediction models can attain relatively high predictive ability for genetic values for complex traits in the Croatian and ORCADES replication. In this study, only 50,000 features were required to achieve an accuracy of prediction for height within the Croatian data of related individuals analogous to that from the full set of features in the supervised feature selection, as opposed to the 100,000 features reported in a recent study^13^. However, 50,000 in this study, as opposed to 5,000 SNPs reported previously, were required to achieve accuracy analogous to that of the full set in the ORCADES replication data of unrelated individuals. This may have resulted from a lower the span of LD between markers and QTL in the genetically isolated Croatian and ORCADES replication populations compared to the two US base populations analysed in the other studies. Nevertheless, the results from HDL, which is influence by a few loci of moderate effect size, are in agreement with the aforementioned study. Whereby, 10,000 and 1,000 features were required to achieve an accuracy of prediction for HDL within the Croatian data of related individuals and into the ORCADES data of unrelated individuals analogous to that from the full set of features in the supervised feature selection respectively. Based on these results, it appears that feature selection has greater benefits in independent replication populations (where individuals are unrelated to those in the training data) for traits which are influence by loci of moderate effect size. Thus the removal of redundancy at lower marker densities increases LD between markers and QTL, and thus the ability IBS matrix to utilise this signal in G-BLUP.

In feature selection we construct a model by choosing an appropriate model representation and estimating model parameters. Feature selection helps us find a parsimonious representation. This is a daunting task in genomic prediction because of the hundreds of thousands of genetic markers to select from, and introduces an additional layer of complexity in the modelling task^48^. In this study, we have developed a set of five feature selection algorithms which accommodate in the inherent structure of GWAS data; that can be implemented to eliminate the least predictive features from a given list of genetic markers. It is also worth adding that the feature selection in our study was conducted on a genome-wide scale. This is an important difference, as previous studies have used simple ranking P-values from the simple linear regression, i.e. supervised feature selection method scenario 1^3^,^7^,^8^,^9^. The advantage of unsupervised feature selection is that it is independent of the prediction model and the predicted phenotypes, and for this reason needs to be performed once, and then different subsets can be evaluated^48^. Apart from the prediction of BMI in the ORCADES data, the supervised feature selection methods outperformed the unsupervised feature selection method across all three traits in this study. This is because the unsupervised feature selection algorithm used in this study ignored the relationship with the phenotype^48^. Thus while excluding many redundant features, it also excluded many relevant features, particularly at low feature subset densities. The superior performance of unsupervised feature selection in the prediction of BMI in the ORCADES replication data may be because the QTL of small effect associated with BMI in this study, are hard to estimate with high accuracy^49^ given the limited size of the training data^16^,^35^. Thus ranking of SNPs following supervised feature selection in this study may have served only to increase LD among SNPs due to shared trait associations. Whereas, unsupervised feature selection which randomly selected SNPs in this could have increased the degree of linkage equilibrium among SNP subsets, and thus augmented our ability to capture additive-genetic relationships^38^, and hence the transfer of LD signal between SNPs and QTL into the ORCADES data. Although more computationally expensive than unsupervised techniques, the supervised approaches have an advantage of selecting variables associated with the phenotype and take feature dependencies into account (Statnikov *et al.*, 2005; Saeys *et al.*, 2007), which generally results in an improved performance. The four feature selection methods we used performed similarly, in terms of reduced dimensionality and optimisation of prediction accuracy. Indeed, when the overall predictive power of data is reasonably good for most of the predictors and extremely low for a few, such as in the case for height and BMI, ranking of GWAS hits in any of the four supervised feature selection scenarios can be used to eliminate the least predictive effects. The good performance of supervised feature algorithms in this study implies that complex relationship of genetic effect on phenotypes should be taken into consideration for selecting features and building a prediction model.

Bayes C outperformed G-BLUP in the prediction of HDL which is influence by loci of moderate effect size using the full feature set. However, the application of supervised feature selection scenario four within the Bayes C framework did not provide substantial improvement in predictive performance over that from the G-BLUP framework. This is because family structure and population stratification accounts for a significant fraction of the prediction accuracy^34^,^35^,^36^,^50^. Family structure and population stratification by standard protocol are accounted for in GWAS^10^, and hence supervise feature selection in this study. On examination many of the top ranked SNPs from Bayes C when all SNPs were fitted in the model; they had low rank following supervised feature selection. This had little impact on the accuracy of prediction of all three traits within the Croatian data, or the highly polygenic traits height and BMI into the ORCADES data, for which the primary source of genetic signal is from relationship information. However, for the prediction of HDL; for which the principal genetic signal is associated with LD between the SNP markers and QTL; there was in substantial downward bias in the accuracy with increasing feature density when predicting into the ORCADES data. This is because the few SNPs that precisely measured family structure and population stratification in Bayes C had low rank following feature selection. Indeed, when the top 5,000 ranked were fitted concurrently with the lowest ranked 63,537 feature selected markers a mean prediction accuracy of 0.142 (95% CI = 0.130-0.154) across the ten test folds was obtained for the prediction of HDL into the ORCADES data. This does not represent a failure of the feature selection method per se, but instead that the same phenotype definition needs to be used in feature selection and application of selected feature subsets.

Computing time for Bayes C increased linearly with the number of markers fitted in the model in this study. In human genetics, a number of bio-bank resources have recently become available, such as Generation Scotland^51^. The MCMC methods, such as Bayes C, are computationally too demanding to apply to half a million or more SNPs and many thousands of training records^52^ associated with these resources – at least when used with Gibbs sampling as in this paper. Feature selection in the G-BLUP frame work therefore provides a flexible and more efficient alternative to computationally time consuming Bayes C for traits that are influenced by loci of moderate effect size. Based on these results, we recommend the application of supervised feature selection within the G-BLUP framework (at least as a useful benchmark) due to its competitive performance and scalability to large datasets.

The results from this study have shown that feature selection significantly improved prediction accuracy above that achieved from the full set of features for height, but not for HDL and BMI when the test samples were drawn from the same cohort as the training samples. One possible reason for the lack of significant improvement of prediction accuracy over the full model for HDL and BMI is that the model does quite well on most of the data sets tested here, leaving little room for improvement. When test samples are drawn from a different unrelated cohort, a more significant improvement might be observed. Indeed the algorithms that used supervised feature selection scenario which ranked SNPs based on inter haplotype block based conditional P-values achieved significantly higher accuracy of prediction for HDL into the unrelated ORCADES dataset than the when the full set of features were applied, whereas, the full feature set was required to optimise prediction of BMI into the ORCADES data. In this study we developed five feature selection methods to handle the structure of GWAS data and to integrate information obtained from established genetic epidemiologic methods in combination with two whole genome regression approaches from animal breeding. It must be stated nonetheless, that a long list of methods have been proposed and used for dimensionality reduction^48^,^53^ and whole-genome regression^13^,^14^,^15^. Their application is beyond the scope of this study. Future work should address other combinations of dimensionality reduction methods with high-dimensional models such as LASSO, elastic net and SVM^13^, GGMs^14^, SIS^15^ and their extensions. Combinations of environmental data with multiple types of omics should be investigated. Additionally, more advanced sampling algorithms^54^ may be considered for improving computational efficiency of Bayesian methods.

## Conclusion

In this paper we concentrated on the predictive performance of five different feature selection algorithms that specifically accommodate the inherent structure of GWAS data for three different traits using three different modelling strategies from animal breeding. In concordance with the infinitesimal trait architecture of height and BMI, that approximates the infinitesimal, the sparse learning Bayes C method and G-BLUP preformed similarly, achieving significantly higher prediction accuracy than the traditional linear regression of GWAS meta-analysis hits. However, as expected Bayes C and linear regression of GWAS meta-analysis hits achieved higher accuracies than G-BLUP in the prediction of HDL which is influence by a few loci of moderate effect size in the ORCADES replication data. Nevertheless, irrespective of the architecture of the traits understudy, the whole-genome regression methods evaluated yielded relatively low accuracies ranging from 0.11-0.26 and 0.02-0.14 when predicting within and into population data respectively. It remains to be determined whether significant increases in sample size, and/or alternative dimensionality reduction or whole-genome regression methods will yield substantial gains in prediction accuracy. The five feature selection algorithms introduced have shown that consideration of the complex relationship of genetic effects on phenotypes reduces the dimensionality of feature space, with little or no loss in predictive performance. Finally, that feature selection in the G-BLUP frame work provides a flexible and more efficient alternative to computationally time consuming Bayes C for less polygenic traits.

## Methods

### Data

The five populations used were CROATIA-Vis, CROATIA-Komiža, CROATIA-Korčula, CROATIA-Split and ORCADES and comprise healthy adult volunteers from the Croatian towns of Vis and Komiža on the island of Vis, the island Korčula, the urban city of Split and from the northern isles of Orkney (Orkney Complex Disease Study, ORCADES, Scotland, UK^55^) respectively. Measurements of height, BMI and HDL cholesterol were obtained for all study participants. The genotypes in this study were generated using a dense Illumina SNP array. In total, 263,357 autosomal SNPs were common across the Croatian samples. The ORCADES study array had 260,562 SNP markers in common with the Croatian arrays. The non-genotyped SNPs in the ORCADES data were imputed using IMPUTE version 2 (IMPUTE2^56^). Following quality control, there were 2,996 phenotypic records available for inclusion in the analysis; 388 from Vis, 509 from Komiža, 816 from Korčula, 473 from Split and 810 from the ORCADES population. Further details on these data are available in the supplementary information (see Supplementary Methods 1).

### Cross-validation

Cross-validation was used to evaluate the ability of a model to predict unobserved phenotypes within the Croatian population data samples. The phenotype data was divided randomly into 10 separate folds of roughly equal size (see Supplementary Methods 2 for further details). To assess the ability of the models trained on Croatian data to predict into a different population, we applied them to predictions in the ORCADES replication cohort. The performance measures reported in this study are the averages of the ten estimates obtained for the 10 folds.

### Prediction performance measure

The performance of each prediction method was assessed based on the correlation between predicted and observed phenotype^47^ in the test and replication data. The theoretical expectation for accuracy^9^ was estimated as (1–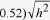 and 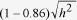 in the Croatian data and ORCADES replication data respectively.

### Population stratification

To eliminate the part of the phenotypic signal that may be associated with large scale population structure the first 20 PCs represented the 5 × 4 possible genetic geographical clines among the five populations in this study were obtained (see Supplementary Methods 3 for details). An additional four PCs were extracted to represent recent admixture among the Croatian populations. The first 24 PCs were therefore added in the G-BLUP model as covariates to investigate the impact of population structure when predicting genomic values^57^.

### Dhata analysis methods

The prediction model chosen is important in genomic prediction. In this paper we examine the predictive performance of three models. Firstly, a linear QTL model in which trait-specific SNPs from reported meta-analyses were fitted simultaneously. Two whole-genome regression models from animal breeding; that regress phenotypes on whole-genome markers simultaneously were also investigated, a linear model (G-BLUP), in which SNPs are weighted equally, and a non-linear method (Bayes C) in which some SNPs are given greater weight. The three methods were compared in terms of predictive performance for traits varying in genetic architecture within and across populations. Empirical analyses have shown differences between genome-wide prediction methods, with a slight advantage of models performing ‘variable selection and shrinkage’ such as Bayes C for traits with ‘large effect QTL’. G-BLUP has been shown to perform well for most traits^13^. There are reports in the literature comparing performance of these methods by using all available SNP data within population^16^,^58^. In this study, we have focused on investigating the effects of using all available SNP data and the effects of feature selection within and into populations (i.e. when the replication cohort is known to be of different ancestry. Further details on these data analysis methods are available in the supplementary information (see Supplementary Methods 4).

### GWAS of Croatian Data

These were performed to generate the basic SNP information upon which to base supervised feature selection. For single-marker GWAS, we used a two-step approach referred to as genomic Genome-wide Rapid Association using Mixed Model and Regression^12^ (see Supplementary Methods 5 for details).

### Feature selection

In this study, we developed one unsupervised and the four supervised feature selection algorithms to accommodate the inherent structure of GWAS data, by incorporating information from established genetic epidemiologic methods^11^,^12^. We used one unsupervised and the four supervised feature selection methods in the G-BLUP framework. A single feature selection method was selected and applied in the Bayes C framework.

### Unsupervised feature selection

We selected ten data subsets for each of 100, 500, 1,000, 5,000, 10,000, 50,000, 100,000, 150,000, 200,000, 250,000 sets of markers evenly spaced from a random starting point (N.B. for sets of 50,000 markers or greater there will be overlap between the ten marker subsets). We refer to the subsets of different numbers of marker as subsets of different densities in what follows.

### Supervised feature selection

In supervised feature selection, the SNP association P-values from the GWAS analyses conducted on each of the Croatian training data folds were used to rank SNPs based on their strength of association with the trait.

Density specific data sets were selected based on the GWAS rank of the SNPs. The performance of prediction models needs to be evaluated on independent test datasets used neither for training the models nor for selecting predictors used by the models. The remaining data is then used for training and performing model selection^59^. To assess the importance of cross-validation in supervised feature selection, we also selected a subset of features using the whole dataset. We then compared the accuracy and performance to those from the model in which the features were cross-validated. To explore the bias in accuracy in relation to the fraction of the test set that was also in the training set, we compared the upward bias in prediction accuracy in test data sets comprising of 1.25%, 2.5%, 5% and 10% of the Croatian data set when test data was used during supervised feature selection.

For the first selection scenario SNPs were selected solely based on their GWAS P-value rank in the training data, ignoring feature dependencies (i.e. LD). Three additional supervised techniques attempted to account for feature dependencies. In scenario two the median marker interval was calculated in the complete genotype data set. The markers were then ranked based on GWAS P-values, and the top hit selected. The selected hit and all markers within the median marker interval were then removed from the selection panel. The process was then iterated until no markers remained for selection. The density specific data sets were then selected based on the GWAS rank of the selected SNPs. In scenario three, the markers were ranked based on GWAS P-values, and the top hit selected. The selected hit and all markers within the median marker interval were then fitted as covariates in a linear model, and conditional P-values extracted. The order in the model was based on GWAS rank. The selected hit and all markers within the median marker interval were then removed from the selection panel. The process was then iterated until no makers remained for selection. The SNP subsets of different sizes were then selected based on intra-interval conditional association P-values. In scenario four, we identified haplotype blocks with PLINK software (version 1.07^11^, following the default procedure in Haploview^60^). All SNPs were partitioned into haplotype subsets. Intra-block SNPs were simultaneously fitted as covariates in a linear model, and conditional P-values extracted. Model order was based on GWAS rank. The density specific data sets were then selected based on the intra-block conditional association P-values.

### Ethics statement

All the Croatian cohorts received ethical approval from the Ethics Committee of the Medical School, University of Split and the NHS Lothian (South East Scotland Research Ethics Committee). The ORCADES study received ethical approval from the NHS Orkney Research Ethics Committee and North of Scotland Research Ethics Committee. All studies conformed to the ethical guidelines of the 1975 Declaration of Helsinki and were approved by appropriate ethics boards, with all participants signing informed consent prior to participation.

## Author Contributions

Grant holders: C.S.H., R.P.-W., P.N. and F.A. Study design: M.L.B., C.S.H., R.P.-W., P.N. and F.A. Provision of the data: C.H., I.R., H.C., A.F.W. and J.F.W. Analytical support: C.S.H., R.P.-W., P.N., F.A. and A.S. Edited the data: M.L.B. Performed the analysis and drafted the manuscript: M.L.B. All authors have read, made changes to and approved the manuscript.

## Additional Information

**How to cite this article**: Bermingham, M.L. *et al.* Application of high-dimensional feature selection: evaluation for genomic prediction in man. *Sci. Rep.* **5**, 10312; doi: 10.1038/srep10312 (2015).

## Supplementary Material

Supplementary Information

## Figures and Tables

**Table 1:** Summary statistics of heritability estimates (standard error in parentheses) for body mass index (BMI), high density protein and height from genomic best linear unbiased prediction (G-BLUP) and Bayes C across the training folds, following tenfold cross validation of the Croatian data.

**Table 2:** Prediction accuracy estimates with 95% confidence intervals, and non-parametric Wilcoxon test P-values from the comparison of estimates from quantitative trait loci (QTL) linear model, genomic best linear unbiased prediction (G-BLUP) and Bayes C using all 263,357 markers within the test data sets, following 10 fold cross validation of the Croatian data and into ORCADES (UK) replication data.
