# Supplementary material for: Application of high-dimensional feature selection: evaluation for genomic prediction in man
Source: Sci Rep. 2015 May 19;5:10312. doi: 10.1038/srep10312 (PMC4437376; doi:10.1038/srep10312)
Supplement: Supplementary Information [file srep10312-s1.doc]

[**Application of high-dimensional feature selection**](http://apps.webofknowledge.com/full_record.do?product=UA&search_mode=GeneralSearch&qid=5&SID=P2IeM@FgElI4@HAClEO&page=1&doc=7&cacheurlFromRightClick=no)**: evaluation for genomic prediction in man**

(Revised for Scientific Reports)

# M.L. Bermingham^1^, R. Pong-Wong^2^, A. Spiliopoulou^1^, C. Hayward^1^, I. Rudan^3^, H. Campbell^3^, A.F. Wright^1^, J.F. Wilson^3^, F. Agakov^4^, P. Navarro^1^ and C.S. Haley^1,2^

^1^MRC Human Genetics Unit, MRC Institute of Genetics and Molecular Medicine, University of Edinburgh, Edinburgh, EH4 2XU, Scotland, UK.

^2^The Roslin Institute and Royal (Dick) School of Veterinary Studies, University of Edinburgh. , Easter Bush, Midlothian, EH25 9RG, Scotland, UK.

^3^Centre for Population Health Sciences, Medical School, Teviot Place, Edinburgh, EH8 9AG, , Scotland, UK.

# ^4^Pharmatics Limited, Edinburgh Bioquater, Edinburgh, EH16 4UX, , Scotland, UK.

* To whom correspondence should be addressed.

Mairead Bermingham,

MRC Human Genetics Unit,

MRC IGMM,

University of Edinburgh,

Edinburgh,

EH4 2XU,

UK.

Email: mairead.bermingham@igmm.ed.ac.uk

Phone: +44 131 3322471

# Supplementary Methods (SM)

## 1. Data

### Study participants

The data in this study stem from five different population-representative cohorts. All studies include a comprehensive collection of data on family structure, lifestyle, blood samples for clinical chemistry, RNA and DNA analyses, medical history, and current health status. All participants gave their written informed consent[^1^](#_ENREF_1). A brief summary of each population is provided below:

1. The Croatia-Vis study includes 960 Croatians recruited from the villages of Vis and Komiza on the Dalmatian island of Vis, Croatia between 2003 and 2004. This cohort included 402 men and 558 women aged between 18 and 93 years.
2. The Croatia-Korcula study includes 897 Croatians from the Croatian Dalmatian islands, recruited in 2007. This cohort included 325 men and 572 women with an age between 18 and 98 years[^1^](#_ENREF_1).
3. The Croatia-Split study recruited 500 individuals from the Croatian urban city of Split between 2008 and 2009. The cohort included 213 males and 287 females aged between the ages 18 and 85[^4^](#_ENREF_4).
4. The Orkney Complex Disease Study (ORCADES) includes a subset of 854 individuals recruited from the Scottish Orkney Islands, between 2005 and 2011[^5^](#_ENREF_5). The study comprised of 400 men and 454 women aged between 17 and 92 years.

All population studies recruited individuals within a community irrespective of any specific phenotype. Blood samples were collected, biochemical and physiological measurements taken and questionnaire data for medical history as well as lifestyle and environmental exposures collected following similar protocols. These populations have all participated in many other GWAS of medically relevant quantitative traits.

### Genotyping and quality control

The DNA samples were genotyped according to the manufacturer's instructions using a dense Illumina SNP array, HumanHap 300v1 for Vis, a mix of HumanHap 300v2 and 370CNV-Quad for ORCADES, 370CNV-Quad for Korčula and 370CNV-Quadv3 for Split, following the manufacturer's standard recommendations. Analysis of the raw data was done in the BeadStudio software with the recommended parameters for the Infinium assay and using the genotype cluster files provided by Illumina. Individuals with a call rate below 97% and SNPs with a call rate below 98%, deviating from Hard-Weinberg equilibrium (p*_HWE_*>1×10^−6^) or with a minor allele frequency of less than 1% were excluded from the analysis. Following this this quality control step. The three Croatian study arrays had 267,912 SNP markers in common. The ORCADES study array had 260,562 SNP markers in common with the Croatian arrays. The ORCADES genotype data were pre-phased using SHAPEIT2[^8^](#_ENREF_8) and 7,109 non-genotyped SNPs imputed using IMPUTE version 2 (IMPUTE2[^9^](#_ENREF_9), using the 1000 genomes reference panel (Phase I Integrated Release Version 3) (PMCID : 3042601) and local exome sequences (<http://dx.plos.org/10.1371/journal.pone.0068604>). The imputation data for SNPs with imputation quality (r2.info index) ≥0.3 was extracted. The **GCTA** software (<http://www.complextraitgenomics.com/software/gcta/reml_bivar.html>) was used to transform the dosage data to PLINK format. The Croatian and original and imputed ORCADES genotype datasets were merged using PLINK (<http://pngu.mgh.harvard.edu/purcell/plink>). Following imputation, individuals from the ORCADES lacked genotype information from only 241 SNPs, which were coded as missing in the merged dataset. When the r^2^ between a pair of markers exceeded 0.95, only one of these two markers was used to avoid singularity in the matrix in Bayes C analysis. Following quality control, 263,357 SNPs remained for inclusion in the analysis. All editing of the genotype data was conducted in the software package GenABEL ([http://www.genabel.org](http://www.genabel.org/))

### Phenotype data and quality control

Measurements of height, BMI and High-density lipoprotein cholesterol (HDL-C) were obtained from four all four population cohorts from Croatia and one replication population from Scotland, which have been described previously [^10^](#_ENREF_10). Data on stature and weight were combined into a body mass index (BMI=weight in kg/height squared in m^2^) as a measure for assessing obesity. Blood samples were drawn after a 12-hour fast and serum was separated and kept frozen until shipped to the biochemical laboratory. The HDL-C in blood samples from the Croatian samples were quantified by electrospray ionization tandem mass spectrometry (ESIMS/MS) using methods validated and described previously[^11^](#_ENREF_11). Whereas HDL-C in blood samples from Orkney were quantified by enzymatic photometric assays using an ADVIA1650 clinical chemistry analyzer (Siemens Healthcare Diagnostics GmbH, Eschborn, Germany) at the Institute for Clinical Chemistry and Laboratory Medicine, Regensburg University Medical Center, Germany[^1^](#_ENREF_1). The HDL measurements in this study were strongly positively skewed. For this purpose we therefore log_e_-transformed the raw measurements to render them approximately normally distributed. The number of individuals available with all trait measures, was 509 for Komiza, 449 for Vis, 883 for Korčula, 513 for Split and 831 for the ORCADES population samples.

### Pre-adjustment and Transformation of phenotype data.

In practice, phenotypes are affected by non-genetic effects such as those of contemporary groups (e.g., population, sex, or age of individuals). In theory, one can extend prediction model by adding these effects; which allows joint estimation of all effects. When feasible this is the preferred option. Nevertheless, in practice this may not be feasible. Indeed, in this study the Gibbs sampler used to implement Bayes C does not allow for the joint estimation of all effects, because of high computational requirements. In such instances, the common approach is to pre-adjust data for non-genetic effects[^12^](#_ENREF_12). Linear regression was therefore used to adjust each trait for age, age^2^, and sex within each of the five populations separately. Extreme outliers (those with residual values more than three standard deviations from the mean) were removed for each trait. The residuals were then transformed to normally distributed *z*-scores. The number of individuals available for inclusion in the analysis with all three trait measures was 509 for Komiza, 388 for Vis, 816 for Korčula, 473 for Split and 810 for the ORCADES studies following quality control at the phenotype level.

## 2. Cross validation

The cross-validation predictions of phenotypes in the first fold were obtained by using individuals in the other 9 folds as training data to predict the phenotype of the individuals in fold 1, and obtain an estimate of the prediction error. This process was repeated for each of the 10 folds, to yield a whole set of cross-validation predictions
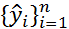
 that were compared to the observed phenotypes
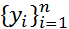
 to assess predictive ability.

The training and test sets in the Croatian data consisted of between 1967 and 1971 individuals and 215 to 219 individuals, per fold, respectively. For prediction into ORCADES the test set consisted of the entire sample of 810 individuals, whereas within the ORCADES sample training and test sets consisted of 729 and 81 individuals per fold, respectively (Table SM1).

# **Table SM1: The number of individuals with phenotypes for height, high density lipoproteins and body mass index (BMI) in the entire data set, the training data, and Croatian and ORCADES (UK) replication test data.**

| **Data** | **Training** | | **Test** | |
| --- | --- | --- | --- | --- |
| **Study** | Croatian | ORCADES | Croatian | ORCADES |
| **Cross validation fold** | **Prediction with in the Croatian and into ORCADES data** | | | |
| **1-9** | 1,967 | - | 219 | 810 |
| **10** | 1,971 | - | 215 | 810 |
|  | **Prediction with in the Croatian and ORCADES data** | | | |
| **1-9** | 1,967 | 729 | 219 | 81 |
| **10** | 1,971 | 729 | 215 | 81 |

## 3. Population stratification.

To eliminate the part of the phenotypic signal that may be associated with large scale population structure, the IBS coefficients between pairs of individuals i and j were estimated from genotype data as follows


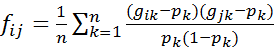
(Formula 1)

where g_ik_ is the genotype of the i-th individual at the k-th SNP (coded as 0, 1/2 and 1, for minor allele homozygote, heterozygote and common homozygote, respectively), p_k_ is the frequency of the major allele, and n is the number of SNPs used for kinship estimation in the statistical package GenABEL[^13^](#_ENREF_13). The genomic relationship matrix was then calculated as twice the kinship matrix.The ancestry principal components (PCs) were obtained following multidimensional scaling of identity-by-state distances using GenABEL’s *“mds”* function[^13^](#_ENREF_13). The first 20 PCs represent the 5×4 possible genetic geographical clines among the five populations in this study. The four additional PCs were extracted to represent recent admixture among the Croatian populations. The first 24 PCs were therefore added in the G-BLUP model as covariates to investigate the impact of population structure when predicting genomic values[^14^](#_ENREF_14).

## 4. Data analysis methods

### Quantitative trait loci (QTL) linear model

For each trait, the retained meta-analysis SNPs were fitted as explanatory variables in a quantitative trait loci (QTL) linear model to estimate allele substitution effects, phenotypes *,*
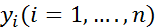
*,* are modelled as the sum of a genomic value,
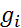
, and a model residual,
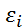
; that is,
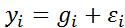
*,* where *ε_i_~*
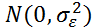
. The predicted phenotype g_i_ for each individual in the test data was calculated as a linear combination of *m* SNPs as:
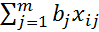
*,* where b_j_ is the estimated effect of allele at locus j and x_ij_ is the number of reference alleles of individual *i* at locus *j*. The proportion of phenotypic variation explained by the SNPs was determined from the unbiased estimator
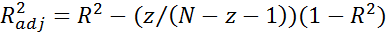
, where *R^2^* is the coefficient of determination of the regression fitting a module of *z* SNPs and *N* phenotypic records[^15^](#_ENREF_15).

In addition, to determine the predictive ability of the baseline SNPs in the G-BLUP framework, relationship matrices using reported trait specific meta-analysis SNPs the were also generated, using the *“ibs”* function (i.e. formula 1, above) of GenABEL[^13^](#_ENREF_13). It must be noted that the prediction accuracy reported from these analyses may have been biased upwards; as the ORCADES, CROATIA-Korčula, CROATIA-Komiža, and CROATIA-Vis, and ORCADES and CROATIA-Komiža, CROATIA-Vis population data samples under study contributed to the meta-analyses used to inform the selection of hits for height and BMI, and HDL respectively.

### Genomic best linear unbiased prediction (G-BLUP)

The G-BLUP model is used to predict genomic values for each trait as: y = Wα+ u + e, where W is a matrix of the first 24 principal components from the IBS matrix, α is a vector of regression coefficients of the principal components, u is the vector of genomic values and e is the vector of residuals. The following distributions are assumed: g~
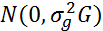
 and e~
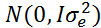
, where *G* denotes a genomic relationship matrix (GRM), and *I* an identity matrix. The genomic heritability was calculated as the ratio:
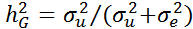
. The linear mixed model was fitted and genomic heritability estimated using residual maximum likelihood (REML) in ASReml[^16^](#_ENREF_16).

Singularity problems arose in the average information matrix when a reduced number of selected SNPs were used in the computation of the GRM. The most common reason for singularities is that the model has been overspecified. Overspecification will occur in a direct product of two unconstrained variance matrices, when there is no information in the data on a particular component. In this instance, the singularities were arising in in the average information matrix, with the model failing as the residual component became too small. The best action is either to revise the variance model so that the ambiguity is removed, or to fix one of the parameters in the variance model so that the model can be fitted[^16^](#_ENREF_16). We resolved this issue by fixing the residual variance to be 0.01, and modelling individual variance as a random effect in the model.

### Bayes C

Bayes C applies the following model: y =
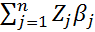
 + e, where Z_j_ is an indicator variable for SNP *j*, and *β_j_* is the allele substitution effect associated with SNP j, and *n* is the number of SNPs. The prior distribution for SNP effects was a mixture of two normal distributions, of two densities: one with small variance (the spike) and one with large variance (the slab). The general form of these mixtures is as follows:
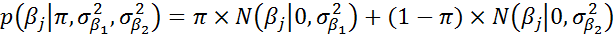
. Where π is the proportion of SNPs that have an effect on the trait,
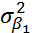
 and
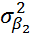
are the variance parameters. Bayes C analyses were implemented using Gibbs sampling in custom made software[^18^](#_ENREF_18). We started by using a fully Bayesian approach with a non-informative beta (1,1) prior for the estimation of π. However, there were problems associated with convergence of the Markov chain Monte Carlo (MCMC) chains resolved by using a weakly informative right-skewed beta (2, 3) prior for π. The variance parameters were estimated from the data using flat inverse-gamma prior distributions in all analyses.

A MCMC chain with 2 million iterations was run for each analysis. The first 500,000 iterations were burn-in. Subsequently, 50,000 realisations were collected, each separated by 30 iterations between consecutive realisations and parameters estimated from these realisations. The genomic values for each individual were calculated as:
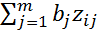
, m is the number of SNPs, b_j_ is the posterior mean effect of allele at locus j and z_ij_ is the number of reference alleles of individual i at locus j. The accuracy was calculated as the correlation of estimated genomic values and the observed phenotype of individuals in the test, and replication data sets.

The convergence of each chain was assessed with the R CODA package [^19^](#_ENREF_19). Analysis of two in dependent MCMC chains with the Gelman–Rubin function in CODA, produced a mean statistic of 1.00 (maximum 95% upper confidence limit of 1.20) for genomic values over cross-validation runs for all three traits under study, suggesting that all chains approximately converged on the same target distribution. Additionally, the chains of genomic effects for each individual were assessed in terms of their autocorrelation, and the effective sample size (ESS) for each genomic effect was calculated [^20^](#_ENREF_20). Based on 1000 realisations, autocorrelations for all genomic breeding values were very close to zero, and the average ESS was ~ 950 across all traits and cross**-**validation runs (Table SM2).

## 5. Genome wide association study (GWAS)

For single-marker GWAS, we used a two-step approach referred to as genomic Genome-wide Rapid Association using Mixed Model and Regression[^13^](#_ENREF_13). In the first step we used the polygenic function in GenABEL package for R[^13^](#_ENREF_13) to fit the model y = u + e, where u is the vector of genomic values and e is the vector of residuals. The following distributions are assumed: g~
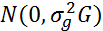
 and e~
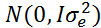
, where *G* denotes a genomic relationship matrix (GRM), and *I* an identity matrix. In the second step, the estimated residuals from step 1 were used for association analysis of each SNP using a linear regression model
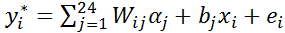
, where
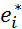
is the estimated residual for the *i^th^* individual,
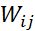
 the *j^th^* principal component score for the *i^th^* individual,
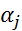
is theregression coefficients of the *j^th^* principal components, x_i_ is the genotype of the i^th^ individual at the marker under study, b_j_ is the effect of j^th^ SNP and e_i_ is the random residual for the i^th^ individual using qtscore function of the GenABEL package for R[^13^](#_ENREF_13).

**Table SM2: The convergence diagnostics of Markov chain Monte Carlo (MCMC) genomic values: the mean (range in parentheses) autocorrelation (r) within chains of estimated genomic values at lag 1, 5, 10, 50, 100 and 100, the mean effective sample size and Monte Carlo error of total genetic effects, average over all 2,966 individuals in the Croatian plus ORCADES data, across the 10 cross-validation runs for estimate for height, high density lipoproteins (HDL) and body mass index (BMI).**

|  | | **Height** | **HDL** | **BMI** |
| --- | --- | --- | --- | --- |
|  | Lag | Auto correlation (r) | | |
|  | 1 | 0.03_(7.11E-08,0.13)_ | 0.03_(7.99E-07,0.13)_ | 0.03_(1.09E-07,0.17)_ |
|  | 5 | 0.03_(2.04E-07,0.13)_ | 0.03_(1.96E-07,0.13)_ | 0.03_(8.25E-07,0.13)_ |
|  | 10 | 0.03_(8.83E-07,0.13)_ | 0.03_(3.41E-06,0.12)_ | 0.03_(2.90E-07,0.13)_ |
|  | 50 | 0.02_(1.97E-06, 0.13)_ | 0.02_(2.27E-07,0.12)_ | 0.02_(1.85E-06,0.12)_ |
|  | 100 | 0.02_(4.94E-07,0.14)_ | 0.02_(2.39E-07,0.13)_ | 0.02_(8.36E-07,0.12)_ |
|  | 500 | 0.02_(1.30E-06,0.10)_ | 0.02_(2.43E-06,0.10)_ | 0.02_(3.40E-06,0.09)_ |
| **Effective sample size** | | 949_(708,1000)_ | 950_(770,1000)_ | 951_(776,1000)_ |
| **Monte Carlo error (%)** | | 0.11_(0.01-0.14)_ | 0.11_(0.10-0.13)_ | 0.11_(0.10-0.13)_ |

**Supplementary Results (SR)**

## 1. Population stratification

Population stratification as a function of the first to fourth principal components of the IBS matrix is shown in Fig. SR1. Of the five populations, Korčula, Komiža and ORCADES populations are clearly distinguishable on the basis of their IBS relationship. The standard G-BLUP model that assumes a homogenous population had higher accuracy than G-BLUP-PC when predicting within the Croatian datasets, however the improvement in prediction was marginal. Conversely, GBULP-PC surpassed G-BLUP in predicting phenotypes into the ORCADES data set. These results are consistent with the conjecture that G-BLUP maybe capturing environmental effects (and/or additive effects) that are extracted by the ancestry principal components (Table SR1-3). Correction for population stratification had only a marginal effect on accuracy within the Croatian population, while improving predictive performance in the unrelated replication population. For this reason, the results from the G-BLUP-PC model will be explored from this point forward, and will henceforth be referred to as G-BLUP. Correction for population substructure in supervised feature selection in the G-BLUP framework had little impact on predictive performance when prediction within the Croatian, or into the ORCADES data. However, at higher feature densities there was a slight reduction in the predictive performance of height and HDL when predicting within the Croatian data, and a marginal improvement for all three trait when predicting into the ORCADES data (Table SR2). The Gibbs sampling software used in this study did not allow us to explore the impact of, or correct for population substructure within the Bayes C framework in this study.


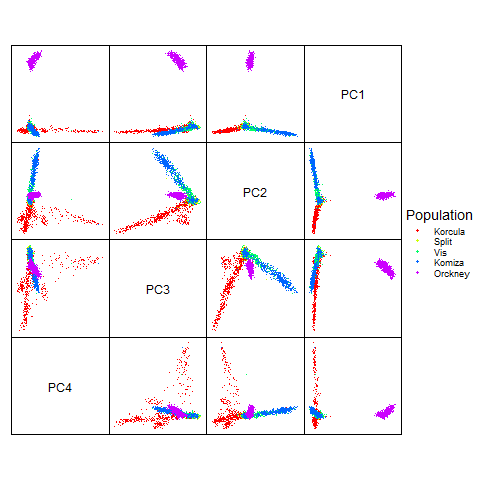

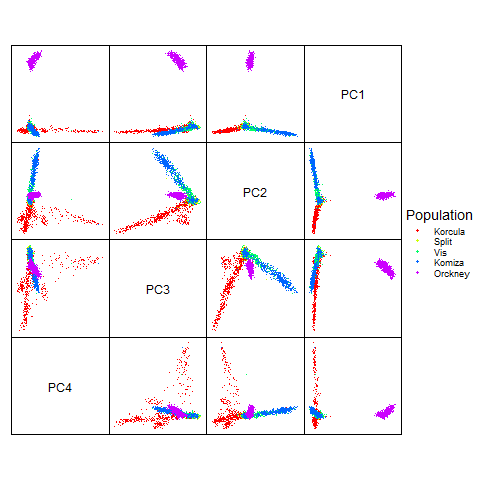

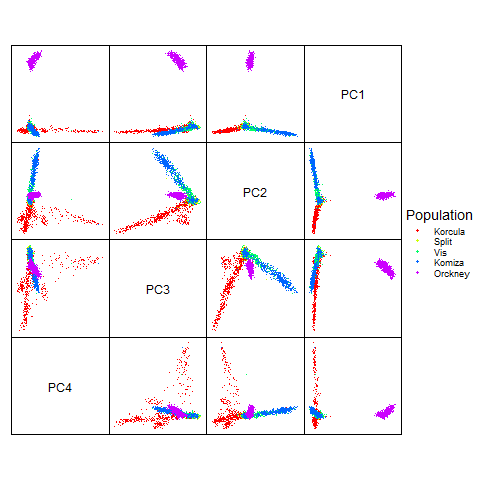

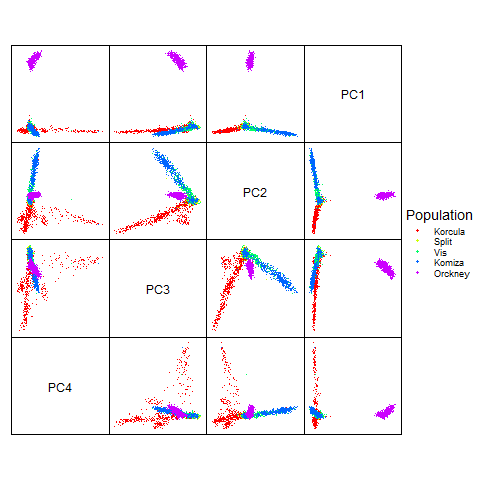


**Supplementary Fig. SR1:** Scatter plots of the first four principal coordinates from multidimensional scaling analysis for genetic matching across the five populations under study. The plots suggest moderate evidence of population stratification among the Croatian isolate populations, and a high level of population stratification between the four Croatian populations and the ORCADES (UK) replication population.

**Table SR1:** Heritability estimate for height, high density lipoproteins (HDL) and body mass index (BMI), with and without correction for population substructure using the top 24 principal coordinates (PCs), using phenotype data from the entire dataset (Croatian plus ORCADES data), the Croatian data, and the summary statistics across training folds following tenfold cross validation of the Croatian data.

| **Data** | **Croatian + ORCADES** | | **Croatian** | | | |
| --- | --- | --- | --- | --- | --- | --- |
|  | **All data** | | **All data** | | **Tenfold cross validation** | |
|  | No PC | 24 PC | No PC | 24 PC | No PC | 24 PC |
| **Height** | 0.826_(0.040)_ | 0.834_(0.040)_ | 0.805_(0.056)_ | 0.796_(0.058)_ | 0.806_(0.014)_ | 0.797_(0.014)_ |
| **HDL** | 0.492_(0.051)_ | 0.480_(0.053)_ | 0.564_(0.067)_ | 0.563_(0.069)_ | 0.530_(0.008)_ | 0.529_(0.009)_ |
| **BMI** | 0.352_(0.052)_ | 0.375_(0.053)_ | 0.285_(0.068)_ | 0.290_(0.071)_ | 0.283_(0.012)_ | 0.290_(0.013)_ |

**Table SR2: Prediction accuracy (correlation between predicted and observed phenotype) estimates for body mass index (BMI), high density lipoproteins and height, and non-parametric comparison of estimates from the test data sets, following 10 fold cross validation of the Croatian data and into ORCADES replication data, from the quantitative trait loci (QTL) linear model, and genomic best linear unbiased prediction (G-BLUP; using all 263,357 markers) with and without correction for population stratification.**

|  | **Accuracy(r_95%CI_)** | | | **Pairwise comparison** | | |
| --- | --- | --- | --- | --- | --- | --- |
|  | QTL model | G-BLUP | G-BLUP+PC | P-value^1^ | P-value^2^ | P-value^3^ |
| **Croatian data** | | | | | | |
| **Height** | 0.12_(0.08-0.17)_ | 0.26_(0.22-0.31)_ | 0.24_(0.20-0.28)_ | 0.002 | 0.001 | 0.027 |
| **BMI** | -0.01_(-0.05_-_0.03)_ | 0.12_(0.08_-_0.15)_ | 0.11_(0.07_-_0.15)_ | 0.002 | 0.004 | 0.432 |
| **HDL** | 0.13_(0.09-0.18)_ | 0.19_(0.16-0.22)_ | 0.17_(0.14-0.20)_ | 0.106 | 0.193 | 0.020 |
| **ORCADES replication data** | | | | | | |
| **Height** | 0.06_(0.01-0.11)_ | 0.05_(0.04-0.06)_ | 0.07_(0.07-0.08)_ | 0.232 | 0.275 | 0.002 |
| **BMI** | 0.02_(-0.02-0.062)_ | 0.06_(0.05_-_0.07)_ | 0.08_(0.07_-_0.09)_ | 0.002 | 0.004 | 0.002 |
| **HDL** | 0.16_(0.12-0.20)_ | 0.01_(0.01-0.02)_ | 0.02_(0.01-0.02)_ | 0.002 | 0.002 | 0.010 |

Acronyms: PC: top 24 principal components from multidimensional scaling analysis of the genomic relationship matrix; 95%CI: 95% confidence interval; r: correlation between predicted and observed phenotypes. The 5% significance threshold with Bonferroni correction (for three comparisons) was set to be less than 0.02. The superscripts 1 and 2 denote the P-values from the pairwise Wilcoxon test between the accuracy results from the QTL linear model, and G-BLUP and G-BLUP+PC respectively. The superscript 3 denotes the P-values from the pairwise Wilcoxon test between the accuracy results from G-BLUP without and with the PC.

## 2. Computational load

The computational time required for a single training fold analysis in the G-BLUP method was 0.017 days whereas, the computational requirement for Bayes C (based on a chain length of 1,500,000 realisations) 3,854 times greater, at 64.11 days. Computing time increased linearly as feature density increased from 100 to 150,000 SNPs (e.g. R^2^= 0.98; p-value= 2.02e-06; training fold 1 for height). It has to be stated however, we have not investigated what would have happened if the MCMC were stopped earlier, or if more efficient samplers than Gibbs sampler were used for approximating the inference in Bayes C models. The goal in this study was to ensure that the MCMC approximately converged to the equilibrium distribution rather than speed up convergence of Gibbs sampler.

**Table SR3:** Prediction accuracy (correlation between predicted and observed phenotype) estimates and pairwise comparison of estimated prediction accuracies across the test data sets, following tenfold cross validation of the Croatian and into the ORCADES replication data using the different marker densities selected using a supervised method of feature selection based on ranking of trait specific GWAS P-values estimates from phenotypes pre-corrected for population structure using the genomic identity by state matrix (IBS)[^21^](#_ENREF_21) versus the accuracy estimated for height, high density lipoproteins and body mass index specific GWAS P-values form phenotypes pre-corrected using the top 24 principal coordinates from, and the genomic IBS matrix[^22^](#_ENREF_22).

|  | **Croatian data** | | | | | **ORCADES replication data** | | | | |
| --- | --- | --- | --- | --- | --- | --- | --- | --- | --- | --- |
|  | Accuracy(r_95%CI_) | | | Wilcoxon-test | | Accuracy(r_95%CI_) | | | Wilcoxon-test | |
| **Cross validation** | G-BLUP | G-BLUP+PC | | P-values | | G-BLUP | G-BLUP+PC | | P-values | |
|  | Height | | | | | | | | | |
| **100** | 0.05_(0.01,0.09)_ | | 0.07_(0.03,0.10)_ | 0.232 | | 0.04_(0.02,0.06)_ | | 0.04_(0.02,0.06)_ | 0.922 | |
| **500** | 0.09_(0.06,0.13)_ | | 0.09_(0.06,0.12)_ | 0.625 | | 0.04_(0.03,0.05)_ | | 0.03_(0.01,0.05)_ | 0.846 | |
| **1,000** | 0.14_(0.11,0.17)_ | | 0.13_(0.10,0.16)_ | 0.160 | | 0.05_(0.03,0.06)_ | | 0.03_(0.02,0.05)_ | 0.037 | |
| **5,000** | 0.22_(0.17,0.27)_ | | 0.21_(0.16,0.27)_ | 0.193 | | 0.03_(0.01,0.05)_ | | 0.02_(0,0.04)_ | 0.160 | |
| **10,000** | 0.26_(0.21,0.3)_ | | 0.24_(0.20,0.29)_ | 0.105 | | 0.03_(0.02,0.05)_ | | 0.03_(0.01,0.05)_ | 0.770 | |
| **50,000** | 0.27_(0.23,0.32)_ | | 0.26_(0.22,0.31)_ | 0.131 | | 0.06_(0.04,0.07)_ | | 0.07_(0.05,0.08)_ | 0.002 | |
| **100,000** | 0.26_(0.22,0.31)_ | | 0.26_(0.21,0.30)_ | 0.064 | | 0.06_(0.05,0.07)_ | | 0.07_(0.06,0.08)_ | 0.004 | |
| **150,000** | 0.26_(0.21,0.30)_ | | 0.25_(0.21,0.29)_ | 0.020 | | 0.06_(0.05,0.07)_ | | 0.07_(0.06,0.08)_ | 0.004 | |
| **200,000** | 0.25_(0.21,0.29)_ | | 0.25_(0.20,0.29)_ | 0.037 | | 0.07_(0.06,0.08)_ | | 0.07_(0.06,0.08)_ | 0.002 | |
| **250,000** | 0.24_(0.20,0.29)_ | | 0.24_(0.20,0.28)_ | 0.004 | | 0.07_(0.06,0.08)_ | | 0.07_(0.06,0.08)_ | 0.193 | |
|  | High density lipoproteins (HDL) | | | | | | | | | |
| **100** | 0.12_(0.06,0.18)_ | | 0.13_(0.09,0.17)_ | 0.625 | 0.11_(0.09,0.12)_ | | | 0.10_(0.09,0.12)_ | | 0.037 |
| **500** | 0.13_(0.07,0.18)_ | | 0.12_(0.06,0.17)_ | 0.105 | 0.06_(0.05,0.07)_ | | | 0.06_(0.05,0.07)_ | | 0.020 |
| **1,000** | 0.12_(0.09,0.15)_ | | 0.10_(0.07,0.14)_ | 0.275 | 0.05_(0.03,0.07)_ | | | 0.05_(0.04,0.07)_ | | 0.232 |
| **5,000** | 0.18_(0.14,0.22)_ | | 0.15_(0.11,0.19)_ | 0.037 | 0.04_(0.02,0.05)_ | | | 0.03_(0.01,0.04)_ | | 0.193 |
| **10,000** | 0.19_(0.15,0.23)_ | | 0.18_(0.14,0.22)_ | 0.193 | 0.05_(0.03,0.07)_ | | | 0.05_(0.03,0.07)_ | | 0.922 |
| **50,000** | 0.17_(0.15,0.20)_ | | 0.16_(0.13,0.19)_ | 0.160 | 0.03_(0.02,0.04)_ | | | 0.03_(0.02,0.04)_ | | 0.432 |
| **100,000** | 0.18_(0.15,0.20)_ | | 0.17_(0.14,0.19)_ | 0.084 | 0.01_(0.00,0.02)_ | | | 0.01_(0.00,0.01)_ | | 0.027 |
| **150,000** | 0.17_(0.15,0.20)_ | | 0.17_(0.14,0.20)_ | 0.084 | 0.01_(0.01,0.02)_ | | | 0.01_(0.00,0.02)_ | | 0.004 |
| **200,000** | 0.17_(0.14,0.20)_ | | 0.17_(0.14,0.20)_ | 0.037 | 0.01_(0.01,0.02)_ | | | 0.01_(0.01,0.02)_ | | 0.027 |
| **250,000** | 0.17_(0.14,0.20)_ | | 0.17_(0.14,0.20)_ | 0.037 | 0.02_(0.01,0.02)_ | | | 0.02_(0.01,0.02)_ | | 0.492 |
|  | Body mass index (BMI) | | | | | | | | | |
| **100** | 0.04_(0.01,0.07)_ | | 0.03_(-0.01,0.07)_ | 0.625 | 0.01_(-0.01,0.02)_ | | | 0.02_(0.00,0.04)_ | | 0.695 |
| **500** | 0.12_(0.08,0.15)_ | | 0.10_(0.06,0.14)_ | 0.049 | -0.01_(-0.03,0.01)_ | | | 0.01_(-0.01,0.02)_ | | 0.557 |
| **1,000** | 0.10_(0.07,0.14)_ | | 0.10_(0.07,0.13)_ | 0.922 | 0.01_(-0.01,0.02)_ | | | 0.00_(-0.02,0.01)_ | | 0.275 |
| **5,000** | 0.07_(0.03,0.11)_ | | 0.06_(0.04,0.08)_ | 0.695 | 0.03_(0.01,0.04)_ | | | 0.04_(0.02,0.05)_ | | 0.084 |
| **10,000** | 0.06_(0.03,0.10)_ | | 0.07_(0.05,0.1)_ | 0.193 | 0.04_(0.03,0.05)_ | | | 0.04_(0.02,0.05)_ | | 0.922 |
| **50,000** | 0.09_(0.05,0.13)_ | | 0.08_(0.04,0.12)_ | 0.275 | 0.04_(0.03,0.06)_ | | | 0.05_(0.03,0.06)_ | | 0.846 |
| **100,000** | 0.10_(0.05,0.14)_ | | 0.09_(0.05,0.14)_ | 0.375 | 0.04_(0.03,0.05)_ | | | 0.05_(0.04,0.06)_ | | 0.770 |
| **150,000** | 0.10_(0.05,0.15)_ | | 0.09_(0.05,0.14)_ | 0.084 | 0.05_(0.04,0.06)_ | | | 0.05_(0.04,0.07)_ | | 0.006 |
| **200,000** | 0.10_(0.06,0.15)_ | | 0.10_(0.06,0.14)_ | 0.160 | 0.06_(0.05,0.07)_ | | | 0.06_(0.05,0.07)_ | | 0.020 |
| **250,000** | 0.11_(0.07,0.15)_ | | 0.11_(0.07,0.15)_ | 0.160 | 0.07_(0.06,0.08)_ | | | 0.07_(0.06,0.08)_ | | 0.010 |

Statistical significance level was set to be less than 0.05.

**Supplementary Figures**


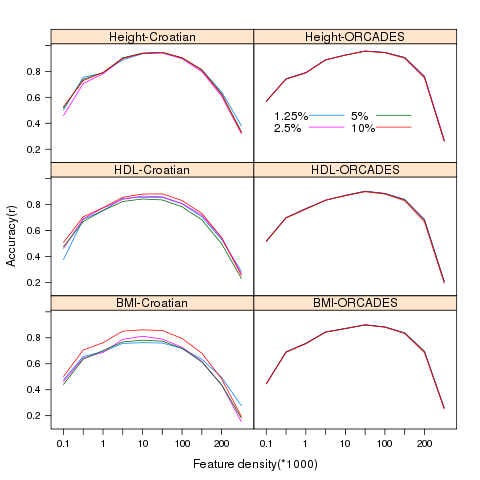


**Supplementary Figure 1:** The mean prediction accuracy (correlation between predicted and observed phenotype) estimated from genomic best linear unbiased prediction across the test data sets, following tenfold cross validation of the Croatian data when the features were not cross-validated (i.e. when feature subset densities were selected based on ranking of trait specific GWAS P-values estimated in all the Croatian data) and into the ORCADES (UK) replication data; when 1.25% (blue line), 2.5% (pink line), 5% (green line) and 10% (red line) of the individuals in Croatian test data were included in the data sets used to train the model. The proportion of Croatian individuals used to train the model included in the test data had no substantial impact on the upward bias in prediction accuracy when the features were not cross-validated.


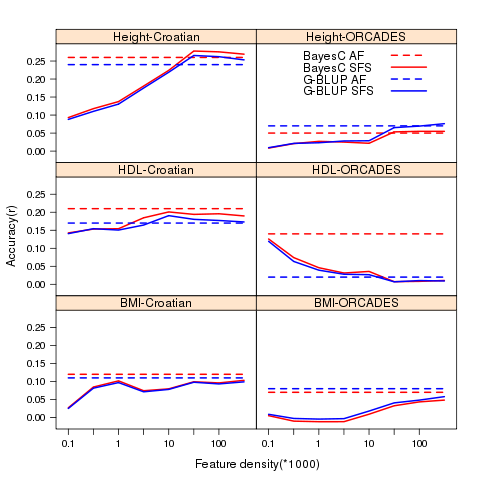


**Supplementary Figure 2:** Average prediction accuracy (correlation between predicted and observed phenotype) for height, high density lipoproteins (HDL) and body mass index (BMI) across the test data sets, following tenfold cross validation of the Croatian data and into the ORCADES (UK) replication data using the different marker densities selected using supervised feature selection (SFS) based on ranking of haplotype-block specific conditional P-values in the Bayes C and genomic best linear unbiased prediction (G-BLUP) frameworks. The broken blue and red lines depict the accuracy results from all features (AF; 263,357 markers) in Bayes C and G-BLUP respectively. The solid blue and red lines depicts the accuracy results across the different feature subset densities following supervised feature selection based on ranking of haplotype-block specific conditional P-values, in Bayes C and G-BLUP respectively.

## References

1 Igl, W. *et al.* Modeling of Environmental Effects in Genome-Wide Association Studies Identifies SLC2A2 and HP as Novel Loci Influencing Serum Cholesterol Levels. *PLoS Genet.* **6**, e1000798 (2010).

2 Rudan, I., Campbell, H. & Rudan, P. Genetic epidemiological studies of eastern Adriatic island isolates, Croatia: objectives and strategies. *Coll. Antropol.* **23**, 531-546 (1999).

3 Vitart, V. *et al.* 3000 years of solitude: extreme differentiation in the island isolates of Dalmatia, Croatia. *Eur. J. Hum. Genet.* **14**, 478-487 (2006).

4 Rudan, I. *et al.* 10 001 Dalmatians" Croatia Launches Its National Biobank". *Croat. Med. J.* **50**, 4-6 (2009).

5 McQuillan, R. *et al.* Runs of homozygosity in European populations. *Am. J. Hum. Genet.* **83**, 359-372 (2008).

6 Repapi, E. *et al.* Genome-wide association study identifies five loci associated with lung function. *Nat. Genet.* **42**, 36-44 (2009).

7 Vitart, V. *et al.* New loci associated with central cornea thickness include COL5A1, AKAP13 and AVGR8. *Hum. Mol. Genet.* **19**, 4304-4311 (2010).

8 Delaneau, O., Zagury, J.-F. & Marchini, J. Improved whole-chromosome phasing for disease and population genetic studies. *Nat. Meth.* **10**, 5-6 (2012).

9 Howie, B. N., Donnelly, P. & Marchini, J. A flexible and accurate genotype imputation method for the next generation of genome-wide association studies. *PLoS Genet.* **5**, e1000529 (2009).

10 Vitart, V. *et al.* SLC2A9 is a newly identified urate transporter influencing serum urate concentration, urate excretion and gout. *Nat. Genet.* **40**, 437-442 (2008).

11 Knežević, A. *et al.* Effects of aging, body mass index, plasma lipid profiles, and smoking on human plasma N-glycans. *Glycobiology* **20**, 959-969 (2010).

12 de los Campos, G., Hickey, J. M., Pong-Wong, R., Daetwyler, H. D. & Calus, M. P. Whole-genome regression and prediction methods applied to plant and animal breeding. *Genetics* **193**, 327-345 (2013).

13 Aulchenko, Y. S., Ripke, S., Isaacs, A. & van Duijn, C. M. GenABEL: an R library for genome-wide association analysis. *Bioinformatics* **23**, 1294-1296 (2007).

14 Yang, J. *et al.* Genome partitioning of genetic variation for complex traits using common SNPs. *Nat. Genet.* **43**, 519-525 (2011).

15 Allen, H. L. *et al.* Hundreds of variants clustered in genomic loci and biological pathways affect human height. *Nature* **467**, 832-838 (2010).

16 Gilmour, A. R., Gogel, B., Cullis, B. & Thompson, R. ASReml user guide release 3.0. *VSN International Ltd, Hemel Hempstead, UK* (2009).

17 Habier, D., Fernando, R. L., Kizilkaya, K. & Garrick, D. J. Extension of the Bayesian alphabet for genomic selection. *BMC bioinformatics* **12**, 186 (2011).

18 Nadaf, J., Riggio, V., Yu, T.-P. & Pong-Wong, R. Effect of the prior distribution of SNP effects on the estimation of total breeding value. *BMC Proc.* **6**, S6 (2012).

19 Plummer, M., Best, N., Cowles, K. & Vines, K. CODA: Convergence diagnosis and output analysis for MCMC. *R news* **6**, 7-11 (2006).

20 Tierney, L. Markov Chains for Exploring Posterior Distributions. *Ann. Stat.* **22**, 1701-1728 (1994).

21 Aulchenko, Y. S., de Koning, D.-J. & Haley, C. Genomewide Rapid Association Using Mixed Model and Regression: A Fast and Simple Method For Genomewide Pedigree-Based Quantitative Trait Loci Association Analysis. *Genetics* **177**, 577-585 (2007).

22 Price, A. L. *et al.* Principal components analysis corrects for stratification in genome-wide association studies. *Nat. Genet.* **38**, 904-909 (2006).
